# Imaging of Functional Connectivity in the Mouse Brain

**DOI:** 10.1371/journal.pone.0016322

**Published:** 2011-01-20

**Authors:** Brian R. White, Adam Q. Bauer, Abraham Z. Snyder, Bradley L. Schlaggar, Jin-Moo Lee, Joseph P. Culver

**Affiliations:** 1 Department of Physics, Washington University, St. Louis, Missouri, United States of America; 2 Department of Radiology, Washington University, St. Louis, Missouri, United States of America; 3 Department of Neurology, Washington University, St. Louis, Missouri, United States of America; 4 Department of Anatomy and Neurobiology, Washington University, St. Louis, Missouri, United States of America; 5 Department of Pediatrics, Washington University, St. Louis, Missouri, United States of America; 6 Department of Biomedical Engineering, Washington University, St. Louis, Missouri, United States of America; Indiana University, United States of America

## Abstract

Functional neuroimaging (e.g., with fMRI) has been difficult to perform in mice, making it challenging to translate between human fMRI studies and molecular and genetic mechanisms. A method to easily perform large-scale functional neuroimaging in mice would enable the discovery of functional correlates of genetic manipulations and bridge with mouse models of disease. To satisfy this need, we combined resting-state functional connectivity mapping with optical intrinsic signal imaging (fcOIS). We demonstrate functional connectivity in mice through highly detailed fcOIS mapping of resting-state networks across most of the cerebral cortex. Synthesis of multiple network connectivity patterns through iterative parcellation and clustering provides a comprehensive map of the functional neuroarchitecture and demonstrates identification of the major functional regions of the mouse cerebral cortex. The method relies on simple and relatively inexpensive camera-based equipment, does not require exogenous contrast agents and involves only reflection of the scalp (the skull remains intact) making it minimally invasive. In principle, fcOIS allows new paradigms linking human neuroscience with the power of molecular/genetic manipulations in mouse models.

## Introduction

The development of functional neuroimaging techniques, particularly functional magnetic resonance imaging (fMRI), has revolutionized human cognitive neuroscience [1]. However, while these advances have transformed the ways that researchers study human brain function, they also have widened the divide between research in humans and in mouse models. Attempting to replicate human fMRI findings in mice is prohibitively difficult as the small size of the mouse brain necessitates exceptionally high signal-to-noise and spatial resolution. These requirements oblige the use of technically challenging and expensive small-animal-specific MRI scanners with very high magnetic field strengths. Conversely, it is difficult to study fMRI correlates of molecular or genetic manipulations without functional maps in the same animals in which the manipulations are derived. Thus, we propose a new method, functional connectivity with optical intrinsic signal imaging (fcOIS), as a simple bench-top method to perform functional mapping of the mouse cerebral cortex using similar hemodynamic contrast to fMRI, with high resolution, high speed, and at low cost.

Neuroimaging of resting-state functional connectivity [2], [3], [4] is a novel approach which promises to integrate cognitive neuroscience with studies of neurological diseases involving patients and animal models. The discovery that functionally-related areas have correlated neural and hemodynamic activity even in the absence of tasks means that brain networks can be studied even in patients with brain-injury [5], [6], those under anesthesia [7], or in subjects unable to perform detailed cognitive tasks, such as neonates [8], [9]. For example, recent studies highlighted the promise of clinical functional connectivity MRI (fcMRI) by showing evidence of the spatial relationships between the brain regions affected by dementia and resting-state networks [10], [11]. Further examination of the molecular basis for such connections, however, is difficult. While fcMRI has been extended recently to non-human primates [7] and rats [12], [13], [14], [15], [16], with initial success in some clinical models (for example, of stroke [15], [17]), published fcMRI methods have not been extended to mice, which would permit more powerful genetic and molecular approaches.

An alternative method for functional neuroimaging in small animals is optical intrinsic signal imaging (OIS) [18], [19], [20], [21], [22] where changes in reflected light intensity off the surface of the brain are converted to changes in local hemoglobin concentrations. Thus, neural activity can be measured through the neurovascular response in much the same manner as in fMRI. Such systems have demonstrated high spatial resolution [23], spectroscopic sophistication [24], and high speed [25]. Additionally, OIS is easily amenable to integration with other measurements, for example fluorescence imaging (including two-photon microscopy) and electrophysiology [22], [26], [27]. These advantages have been highlighted by OIS studies of neurovascular coupling [28], [29], [30], spontaneous activity [31], and cortical columns [31], [32].

In this paper, we combine resting-state functional connectivity and OIS methods to perform novel functional connectivity optical intrinsic signal imaging (fcOIS) in mice. Using fcOIS, we demonstrate the first functional connectivity maps in mice covering almost the entirety of the convexity (from the olfactory bulb anteriorly to the superior colliculus posteriorly and laterally through primary somatosensory and auditory cortex). Having determined the patterns of functional connections, we show their utility through the use of functional connectivity data to parcellate the mouse cortex into functional areas. This mapping of the spatial arrangement and extent of multiple functional networks yields results in agreement with the expected neuroarchitecture. As fcOIS relies on simple and relatively inexpensive camera-based equipment and requires only the reflection of the scalp (making it minimally invasive), we expect that this method will be a widely useful tool, giving mouse researchers access to functional neuroimaging and allowing human neuroimagers to test hypotheses in standardized mouse models.

## Results

### Functional Connectivity in the Mouse Brain

**Figure 1:**
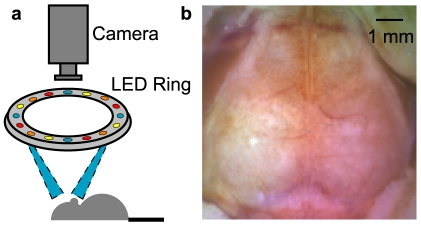
System for fcOIS. (a) Illumination from sequentially flashing LEDs in four different wavelengths (478 nm, 588 nm, 610 nm, and 625 nm) arranged in a ring. Detection by an EMCCD camera is at 120 Hz (30 Hz after decoding of wavelengths). Crossed linear polarizers (not shown for simplicity) prevent artifacts from specular reflection off the skull. (b) A false color image of the mouse cortex generated from the red, yellow, and blue LED channels. The image shows the camera's field-of-view (approximately 1 cm^2^) of the mouse brain with the cerebral cortex visible through the skull from the olfactory bulb to the superior colliculus and far laterally on the convexity. In the corners, one can see the reflected skin flaps. The brain was manually segmented from the image providing a mask for fcOIS analysis.

We mapped functional connectivity using a custom-built high-speed (30 Hz) OIS system (Fig. 1a). This set-up captures a large field of view of the mouse brain visible through the intact skull (Fig. 1b), from which the brain was manually segmented. Data from multiple wavelengths were synthesized using a tissue spectroscopy model to yield time traces of changes in oxy- and deoxyhemoglobin (HbO_2_ and Hb_R_, respectively) at all visible brain locations. Imaging was performed on five mice (all male ND4 Swiss Webster anesthetized with Ketamine/Xyalzine); resting-state data were acquired for at least 15 minutes on each mouse (see Methods for further details of the system and experimental conditions).

Resting-state functional connectivity methods evaluate spatio-temporal correlation patterns in spontaneous brain activity (here viewed indirectly through the neurovascular response) [2], [33], [34]. The connections of a given region (referred to as a seed) can be measured by performing a simple Pearson's correlation analysis between the seed and other cortical locations (either in a seed-to-seed analysis or with every pixel in brain). The original discovery that enabled fcMRI [2] was that functionally-related regions (specifically motor cortex) had correlated low frequency hemodynamics even in the absence of a functional task. In addition, regions in the brain that have opposing functions (for example attention and default-mode regions in the human brain [35]) have resting-state time traces that are anticorrelated. Thus, the examination of resting-state correlation maps (consisting of *r*-values between regions of interest and the rest of the brain) can reveal multiple functional networks. It should be noted that the interpretation of anti-correlations warrants some caution as the removal of a global mean signal enforces the presence of anticorrelated pixels. However, the pattern of the anticorrelations is entirely data-driven and is still of interest [36]. To determine the pattern of functional connections in the mouse brain, we extracted seed time traces from every major cortical region within our field-of-view: right and left visual, somatosensory, motor, frontal, cingulate, and retrosplenial cortices as well as the olfactory bulb and superior colliculus. The coordinates for this analysis were chosen based on the visualized anatomy and expected positions from a histological atlas [37]. At each of these locations, the seed time trace (ΔHbO_2_) was made by averaging over a 0.5 mm diameter circle (approximately 30 pixels) centered at the chosen coordinate.

**Figure 2:**
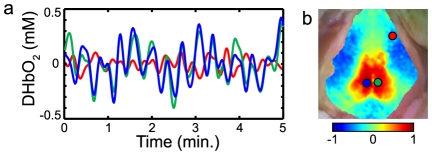
Performing functional connectivity with OIS. (a) Time traces (ΔHbO_2_) for three cortical locations: left retrosplenial (blue), right retrosplenial (green), and right motor (red). The right and left retrosplenial are functionally related and show time traces with high correlation (*r* = 0.88), while the left retrosplenial and right motor cortices are anticorrelated (*r* = −0.47). (b) A functional connectivity map made by correlating the left retrosplenial seed (blue circle) with all other brain pixels. High correlation values show functional related regions, including the right retrosplenial (green circle), while other regions are negatively correlated, including the right motor cortex (red circle). Correlation values near zero are found in functionally unrelated regions (frontal and visual cortices).

**Figure 3:**
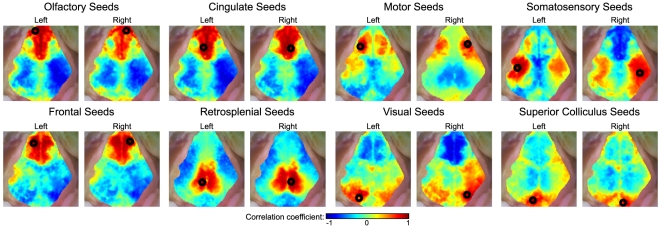
Seed-based fcOIS. Correlation maps for seeds chosen manually using the expected cortical positions of various functional areas (Mouse 1). Seed positions and sizes are shown with black circles. The scale for all correlation maps is from *r* = −1 to 1. Maps are shown overlaid on the “white light” image of the brain. Note the bilateral patterns for all seed locations.

fcOIS correlation analysis revealed distinct resting-state networks. Comparison of seed time traces showed both high correlatations (*r* approaching 1), particularly between contralateral homotopic seeds, and anticorrelations (negative *r*), putatively between functionally-opposed regions (Fig. 2a). An image made by correlating a seed time trace with every pixel in the image thus extends this concept to the construction of a full functional connectivity map (Fig. 2b). Maps for the sixteen seed locations in Mouse 1 showed bilateral functional connectivity patterns: correlations with adjacent cortex as well as homotopic contralateral cortex (Fig. 3). Beyond these connections, there are also very strong correlations between olfactory, frontal, and cingulate cortices, with anticorrelations to somatosensory regions. In between the visual and somatosensory seeds (the location of the mouse's small parietal cortex), there are regions that show correlations with both seeds. And, the retrosplenial cortex shows a very sharp delineation from adjacent cortical areas including anticorrelations with the motor seeds.

Similar patterns are visible in all five mice scanned (Fig. S1). In two mice (Mouse 2 and Mouse 5), the field of view was extended laterally enough to result in putative bilateral auditory correlations (Fig. S2). Furthermore, the functional connectivity patterns are repeatable within the same mouse. Correlation maps made using two consecutive fifteen minute scans in the same mouse (Fig. S3) or maps made on two consecutive days (Fig. S4) show very similar structure. This similarity can be quantified by evaluating the spatial correlation between two consecutive maps using the same seed location. In three mice (2, 4, and 5) data were collected for 30 min. The spatial correlation between maps made from consecutive fifteen minute segments is 0.86+/−0.06 for all cortical seeds. The shapes of the connectivity patterns vary slightly between mice, while the connectivity patterns remain more consistent between multiple scans on the same mouse suggesting that the inter-mouse differences in functional connectivity reflect differences in the shape and position of different functional brain regions.

Seed-based functional connectivity maps are biased by the particular choice of the seed location. To address this issue, we also evaluated a method that is independent of user input. First we constructed the full correlation matrix, which contains correlation values between each pixel and every other pixel in the entire image. Because this produces many patterns that are essentially redundant, we used singular value decomposition (SVD) to find the predominant orthogonal functional connectivity patterns (the first four singular vectors are shown in Fig. S5). The first four ordered singular values represent approximately 67% of the total variance in the pixel-to-pixel correlation matrix, and the first ten singular values represent 82% of the total variance. The resulting first four singular vectors show respectively: (1) a very strong frontal/cingulate network that anticorrelates with the bilateral sensory areas, (2) the bilateral retrosplenial cortex that anticorrelates with the sensory areas, (3) the visual areas and the superior colliculus, and (4) more medial motor areas. Thus, the results of the data-driven SVD-correlation matrix analysis strongly corroborate the seed-based results.

### Parcellation

Having found the resting-state connection patterns, we wanted to use this information to divide the surface of the cortex into its component regions. Such a method will group cortical pixels based on similarities in their functional connectivity and will, ideally, regenerate the map of the expected neuroarchitecture. From the above functional connectivity maps, one can already observe borders around highly correlated regions. However, we desired a method to identify the borders from the maps without need for user input. Thus, we devised an automated parcellation scheme to take intuitive interpretations of the connectivity patterns and recreate them in a data-driven manner (see Methods).

**Figure 4:**
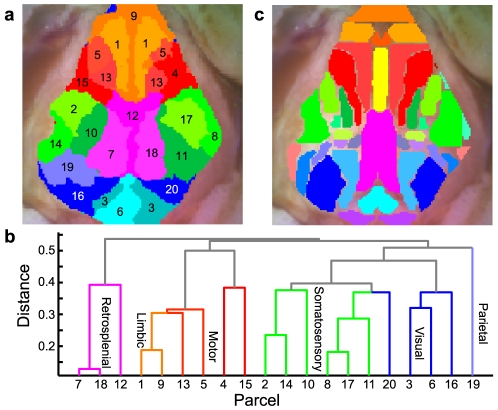
Iterative parcellation of fcOIS data. (a) The results of iterative parcellation using the first twenty singular vectors from the correlation matrix as an initial condition. We see clear delineation of a frontal/olfactory/cingulate (limbic) network (oranges), a motor network (reds), a somatosensory network (greens), a visual network (blue), the retrosplenial cortex (magenta), and the superior colliculus (light blues). Numbers on the parcels are arbitrary designations from the initial condition. (b) Dendrogram showing clustering of the parcels from their correlations. Each terminal branch is a parcel (numbered to match the parcellation image and color-coded based on functional assignments); parcels that are more closely related (i.e., that share similar correlation maps have branches that meet lower on the tree. Note the tight correlations within the frontal network, in turn connecting to first medial and then lateral motor areas. In total, there are main branches for all of the main networks we expect. (c) The Paxinos atlas applied to this mouse brain for comparison with the functional parcellation. (For the names of the different cytoarchitectural regions shown in the atlas, see Fig. S8).

**Figure 5:**
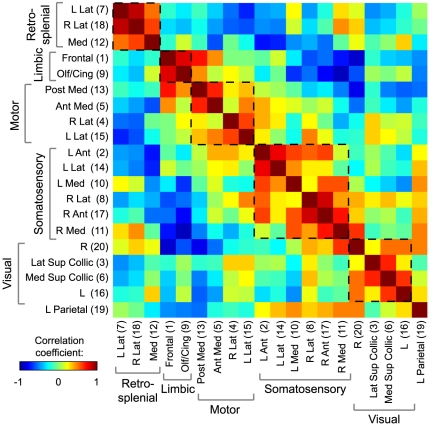
Correlation matrix between parcels after iterative parcellation. Each row and column corresponds to a parcel (labeled with a functional assignment, anatomic location, and a number that matches the scheme in Fig. 5). We see a block-diagonal pattern showing how the clustering has arranged the parcels into networks (dashed boxes shown for added visualization). Off-diagonal elements show the relationships between networks; in particular, note the anticorrelations between frontal and somatosensory and between retrosplenial and motor. The left parietal region correlates with both visual and somatosensory regions. Also note how each somatosensory parcel correlates most highly with its similarly named homologue in the opposite hemisphere.

The parcellation method divides up the cerebral surface into functional zones (Fig. 4a shows the results in Mouse 1) with an organization familiar from our earlier examination of connectivity patterns (frontal/cingular, motor, somatosensory, retrosplenial, parietal, and collicular parcels are all found). These individual parcels can be combined into larger functional regions (Fig. 4b) using a clustering algorithm applied to the correlation matrix This grouping and labeling of the functional structures closely resembles the histological regions defined using the coordinates from a histological atlas (Fig. 4c). Once the clustering has ordered the parcels based on their similarity, larger networks are easily seen in the parcel-to-parcel correlation matrix (Fig. 5). The pattern of regions is robust and an inherent result of the functional connectivity networks and is not a random effect of the parcellation method, as very similar borders can be found using alternate initial conditions (Fig. S6).

**Figure 6:**
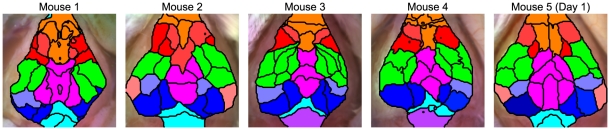
fcOIS Parcellations in multiple mice. Different networks have been color-coded (green for somatosensory; red, motor; orange, frontal/cingulate/olfactory; magenta, retrosplenial; blue, visual; gray-blue, parietal; light blue, superior colliculus; purple, inferior colliculus; pink, auditory). Note that overall the patterns are similar across all the mice though there are slight individual differences in borders of the functional areas.

Performing parcellation analysis on resting-state functional connectivity data from multiple mice yields similar maps (Fig. 6). In every mouse, we can distinguish frontal/olfactory/cingulate, retrosplenial, motor, somatosensory, and visual/superior colliculus networks. Additionally, in some mice, lower regions in the brain stem (putatively inferior colliculus) form their own network uncorrelated with the rest of the networks. Similarly, putative parietal areas are also seen in most of the mice.

Although the exact shape of each region's borders differed between mice, these minor variations are consistent with the slight differences seen in the seed-based correlation maps (Fig. S1). Parcellations performed on two consecutive fifteen minute scans in the same mouse show a consistent division of functional regions (Fig. S7). With this analysis, we can estimate the precision with which we can determine borders. Most of the borders can be localized to within 150 µm. In some regions (between somatosensory and motor and between frontal and motor), the borders are very precise, with an estimated localization uncertainty on the order of 50 µm. In other regions that are more difficult to parcel (e.g., the region where cingulate, motor, somatosensory, and retrosplenial all meet) precision is poorer; the estimated uncertainty is approximately 250 µm.

## Discussion

We have shown (to our knowledge) the first results using optical intrinsic signal imaging to measure functional connectivity, and the first published mapping of functional connectivity in mice with resting-state hemodynamics. The findings of fcOIS were repeatable in time and also robust across multiple mice. These results satisfy our original goals of determining functional connections within the mouse brain in the resting state and of using the patterns of connections to generate a map of functionally distinct parcels. The functional neuroarchitecture found with fcOIS matches our expectations from previous studies in rats, primates, and humans as well as expectations that distinctions between functional regions should correspond to histological patterns [37].

Bilaterally symmetric functional connectivity is a prominent feature of our mapping results in visual, somatosensory, motor, frontal, cingulate, and retrosplenial cortices, as well as the olfactory bulb and the superior colliculus (these being all of the major parts of the brain within our field-of-view; for an equivalent human result see Salvador *et al.* [38]). We did not observe widely distributed anterior-posterior functional connectivity, as typically found in humans (e.g., Fox *et al.* [35]), possibly because large-scale, integrative functional processing is unlikely to occur in the mouse. The frontal, olfactory, and cingulate regions were all highly correlated with each other, and the visual cortices were weakly correlated with the superior colliculus. These patterns resemble those previously found in rats with fcMRI [14]. Similar network patterns were obtained using the data driven SVD analysis. Improved statistical methodology is possible to more fully determine which correlations are the most significant and how their location varies between mice. The standard of practice in human fcMRI is to submit multi-subject datasets to statistical tests of significance using fixed effects [35] or random effects [39] analyses. However, such procedures depend on the availability of a standardized reference head space (known as an atlas) [40], [41]. Once standardized functional OIS atlasing methods for the mouse brain have been agreed upon, these statistical analyses would be straightforward extensions of the work in this paper and would be useful in many fcOIS applications.

Once we were able to demonstrate the presence of resting-state functional connectivity networks in the OIS data, our goal was to use this data to recreate the functional divisions within the mouse cortex and to recreate parcellations found in histological atlases. Our iterative parcellation scheme followed by clustering is able to divide the brain into networks in a data-driven manner. This method robustly parcellates the brain into similar functional regions as are found in the histological atlas [37].

In addition to the sensory and motor cortices, we also found functional connectivity (and associated parcellations) of higher-order cortical areas. Identifying these networks with resting-state neuroimaging is particularly noteworthy as developing task-paradigms to activate “cognitive” regions is difficult in the mouse. The olfactory, frontal, and cingulate cortices are all limbic areas [42], hence, it is expected that they would be highly correlated in the resting state. The functional network including the retrosplenial region most likely represents the murine equivalent of the primate default mode network [7], [43]. As in humans, mouse retrosplenial cortex, an evolutionary older structure, shows strong anti-correlations with more recently developed neocortical regions (e.g., somatomotor and visual cortex). The retrosplenial network in the mouse lacks certain human default-mode network components (e.g., dorsal medial prefrontal and lateral parietal cortex) but this is expected, as these regions are hypothesized to be later evolutionary additions [44].

While, in the present analysis, we have focused on comparisons of large-scale functional distinctions (e.g., between retrosplenial and somatomotor regions), future methodological development could provide robust finer distinctions (e.g., between subdivisions of visual cortex). That such further differentiation might be possible is suggested by the interesting finding that the multiple parcels in somatosensory cortex (as in Fig. 4a) correlate most highly with their putative contralateral homologue. Thus, for example, medial left somatosensory cortex correlates most highly with medial right somatosensory cortex (see Fig. 5).

Several potential improvements of fcOIS correlation mapping technique can be identified. For example, in this paper we used only ΔHbO_2_ as a contrast. While previous functional connectivity studies with optical techniques have shown similar mapping results using different hemoglobin species as contrasts [4], the high resolution, event-related OIS functional mapping literature provides evidence for differences in the spatial extent of functional maps derived from different contrasts (HbO_2_, Hb_R_ or Hb_T_) [45], [46], [47], [48], which should be explored within the resting state. Optical imaging's ability to image multiple contrasts simultaneously can provide estimates of metabolic variables (such as oxygen extraction fraction and cerebral metabolic rate of oxygen) [49]. The role of these parameters in functional connectivity (with their perhaps tighter coupling to the underlying neuronal physiology) remains to be studied.

Additionally, while we used the same functional connectivity frequency band as in previous human and rat studies, the dependence of murine fcOIS on temporal filtering remains a question for future investigation. The use of different frequency bands could potentially capture fast vs. slow correlations that reveal the structure of the brain's information processing, as has been recently attempted in human fcMRI [50]. Additionally, different frequencies might capture information about different vascular compartments, similar to looking at different temporal windows in task-activation studies [47], [48], [51].

Numerous studies have also shown that functional connectivity persists, albeit in modified form, under anesthesia [7], [52]. We chose ketamine/xylazine because it is a relatively simple preparation and therefore well suited to a proof of principle demonstration of the ease of the fcOIS method. However, recent activation studies have shown that neurovascular coupling is more consistent under α-chlorolose [53]. Further, recent fcMRI rat studies have shown improved functional connectivity mapping under α-chlorolose compared to isofluorane and medetomidine [52]. We tested the stability of our fcOIS maps by splitting datasets into separately analyzed halves and observed good reproducibility in individual mice (see Figs. S3 and S7). This consistency suggests stable depth of anesthesia over the duration during which these mice were imaged. Further studies with α-chlorolose may improve the precision of the functional border locations and reduce the modest differences in parcellations observed across imaging sessions (as in Fig. S7).

Future work is also needed to address fcOIS accuracy through direct comparisons to histology. The most direct comparison would be with activation studies using somatosensory (e.g., whisker, forelimb, hindlimb), auditory, or visual stimuli, as in the seminal report of Biswal *et al.* [2]. This evaluation would have the advantage of comparing two hemodynamically derived maps within the same mouse. However, a comprehensive mapping of all functional areas would be considerably involved, and some (such as cingulate) would be difficult to localize with stimulus paradigms.

Alternatively, comparison could be made to histological staining in order to comprehensively define all functional brain regions, which would be the gold standard for quantifying differences in brain organization between mice. In such an analysis, if fcOIS maps were to differ from the histological atlas, steps would be need to be taken to determine whether the divergence was due to variation in the arrangement of cytoarchitecture, noise in the imaging method, or functional connectivity borders differing from histological borders. Although the parameter space for both the optimization and validation of fcOIS is large, these studies will be critical in establishing a firm foundation for fcOIS as a tool for routine mouse neuroscience.

Additionally, a focus of current fMRI research is how closely functional and structural connectivity are connected. In humans, structural connectivity can be assessed only indirectly using diffusion tensor imaging (DTI). While studies comparing fcMRI and DTI have shown reasonable agreement [54], [55], it is difficult for networks to be comprehensively assessed. In mice, neuronal connections could be directly visualized using invasive axonal tracing studies. Such studies, combined with fcOIS could help elucidate the role of multi-synaptic connections in resting-state functional connectivity and how functional networks evolve with the development of structural neural connections.

We expect that advances in MRI technology and methods will eventually allow fMRI-based functional connectivity mapping in mice. However, the need for high-field MRI scanners will most likely restrict its use to dedicated neuroimaging researchers and centers. In contrast, fcOIS provides a combination of high resolution, low cost, and ease of use (a simple intraperitoneal injection of anesthetic and no thinning of the skull) that should enable many laboratories that previously did not consider functional neuroimaging to connect with on-going studies of human disease. One physical limitation of OIS (due to light scattering) is the restriction of the field-of-view to the cortical surface (<1 mm), which precludes direct mapping of deep brain structures (e.g., the thalamus and hippocampus). Thus, we expect the two methods to eventually play a complementary role where interesting results can be found “at the benchside” using fcOIS, and then a subsequent fcMRI study could be done to visualize deep brain structures and compare with high-resolution anatomic scans [56], [57].

In summary, we have demonstrated functional connectivity mapping with OIS in mice. Because we have determined that fcOIS is able to map both functional regions and their connections, this methodology should be a powerful tool for detecting when functional connectivity networks are disrupted (either in the distribution of the neuroarchitecture or in the pattern of connections). Thus, one could examine the functional consequences of disease models including genetic[58] and surgical[59], [60] disruptions. Imaging the development of neurodegenerative disease (e.g., Alzheimer's and Huntington's) in mouse could provide a less circumstantial link between the molecular mechanisms and the tendency for disease to target specific cortical networks[10], [11] providing better insight into both pathophysiology and therapeutic targets[61]. We expect that fcOIS could be a useful tool to connect the intriguing neuroimaging results of human disease obtained through fcMRI with advances in mouse models.

## Methods

### Animal Preparation

All procedures were approved by the Washington University School of Medicine Animal Studies Committee (protocol # 20080216). Male Swiss Webster mice (6–10 weeks of age, 23–32 g, Harlan Laboratories) were anesthetized with a Ketamine/Xylazine mixture (86.9 mg/kg Ketamine, 13.4 mg/kg Xylazine) and allowed 30 minutes for anesthetic transition. Anesthetic effect was verified by ensuring that the animal was not responsive to a hind paw pinch. Once induced, the animal was placed on a heating pad maintained at 37°C (mTCII, Cell Microcontrols) and its head secured in a stereotactic frame using a nose cone and ear bars. The scalp fur was shaved and prepped, and a midline incision was made along the top of the head and the scalp was reflected, exposing approximately 1 cm^2^ of the skull. The skull was kept moist with an application of mineral oil before each scan. Arterial blood pressure from the left femoral artery was monitored using a blood pressure analyzer (Digi-Med, BPA 400a) and measured to be 95±10 mmHg (mean blood pressure +/− standard deviation averaged across mice 2–4; blood pressure data were not available for mouse 1). Scan times were 15, 30, 20, 30, and 30 minutes for mice 1–5 respectively.

### Imaging System

Sequential illumination was provided at four wavelengths by a ring (diameter  = 7 cm) of light emitting diodes (LEDs; 478 nm, 588 nm, 610 nm, and 625 nm; RLS-5B475-S, B5B-4343-TY, B5B435-30S, and OSCR5111A-WY, respectively, Roithner Lasertechnik) placed approximately 10 cm above the mouse's head. For image detection, we used a cooled, frame-transfer EMCCD camera (iXon 897, Andor Technologies) set to acquire via external triggering. The LED ring and the camera were time-synchronized and controlled via computer using custom-written software (MATLAB, Mathworks). To acquire images at a frame-rate well above the heart and respiration rates (∼10 and 2.5 Hz, respectively), we used a full frame rate of 30 Hz, which, with four temporally encoded wavelengths, required running the camera at a frame rate of 120 Hz. To prevent specular reflection from the surface of the mouse skull, crossed linear polarizers were placed just in front of the LEDs and the camera lens. A simplified diagram of the system is shown in Figure 1a.

The secured mouse was placed at the focal plane of both the camera and the LED ring and held in place with a stereotactic holder. The field-of-view was adjusted to be approximately 1 cm^2^ square resulting in a field-of-view that covers the majority of the convexity of the cerebral cortex with anterior-posterior from the olfactory bulb to the superior colliculus (Fig. 1b). Data acquisition at a frame-rate of 120 Hz was made possible by binning on camera (4×4 binning reduced the output image from 512×512 pixels to 128×128 pixels) and spooling the data directly to disc (a 5-minute scan at 120 Hz produces 1.2 GB of data). The resulting pixels were approximately 80 µm×80 µm.

### Image Processing

Image light intensity was interpreted using the Modified Beer-Lambert Law: Φ(t)  =  Φ_0_*exp(-Δμ_a_(t)*L). Here Φ(t) is the measured light intensity, Φ_0_ is the baseline light intensity (with no hemodynamic perturbation), Δμ_a_(t) is the change in absorption coefficient due to changes in blood volume, and L is the path length of the photons in the tissue. With resting-state activity there is no pre-stimulus baseline and instead we normalized relative to the average light intensity: ΔΦ (t)  =  -ln(Φ(t)/<Φ_0_(t)>)  =  Δμ_a_(t)*L. If we are only interested in intensity changes at a single wavelength, then there is no need to correct for the multiplicative constant, L. In order to perform spectroscopy, and recover Δμ_a_(t), we used path length factors calculated using the analytical formula given by Arridge [62] (specifically, Equation 34 in that reference), resulting in differential measures of absorption at different wavelengths: Δμ_a,λ_(t)  =  -ln(Φ_λ_(t)/< Φ_0λ_(t)>)/L_λ_.

We then converted absorption coefficient data to hemoglobin concentration changes using the spectroscopy matrix: the system of equations, Δμ_a,λ_ (t)  =  E_λ,i_ Δ[Hb_i_](t) (where E is the extinction coefficient matrix and *i* runs over hemoglobin species) inverted to find the least-squares solution to the changes in oxy- and deoxy-hemoglobin at each pixel at each time. The hemoglobin extinction values were taken from Prahl [63]. Images in each contrast were smoothed with a Gaussian filter (5×5 pixel box with a 1.3 pixel standard deviation).

To create a false color “white light” image of the mouse brain, the first images from the red (625 nm), yellow (588 nm), and blue (478 nm) LED channels were normalized to a maximum value of one and then stored in the red, green and blue channels of an RGB image (Fig. 1b). This image was viewed in Adobe Photoshop and all regions not corresponding to brain were manually painted white. The image was loaded back into MATLAB and was used to create a brain mask. All further analysis was performed only on those pixels labeled as brain.

### Atlas Construction

To guide seed placement, an atlas of the locations of cortical functional regions (as viewed from a superior projection of the convexity) was constructed using a histological atlas [37]. In every coronal slice, the lateral extent of every cortical area viewable from above was noted, and these coordinates were used to construct polygons surrounding each region. This procedure was repeated using sagittal slices. From these two sets of polygons, a smoothed “consensus” atlas segmentation was produced (Fig. S8).

In the atlas, we noted the position of the junction between the olfactory bulb and cerebrum along the midline and the position of the fissure between the superior colliculus and the cerebrum along the midline (which is also the position of lambda). These two points also were found in the “white light” mouse brain images. Using these two points, the atlas was affine-transformed to brain coordinates; this transform used only one stretch component (the anterior-posterior stretch was also used for the medial-lateral stretch). Then, every pixel in the mouse brain (as defined by the earlier mask) could be assigned to segmented cortical polygons from the atlas (as in Fig. 4c). Note that gaps were purposely left in the atlas segmentation between regions so that brain regions would be smooth. Thus, not every pixel in the OIS image is assigned a putative cortical region.

### Functional Connectivity

Since previous functional connectivity studies [4] with diffuse optical tomography showed similar maps using either HbO_2_, or Hb_R_ contrast, here we used only ΔHbO_2_ data for the connectivity analyses. Data were filtered to the functional connectivity band (0.009–0.08 Hz) following previous human functional connectivity algorithms [35]. While one might expect the frequencies involved in functional connectivity to scale with the size of the animal (as does heart and respiratory rate), studies of fcMRI in rat have used the same frequencies as found in humans [13], [16], [64]. Our results also demonstrate that low frequency fluctuations in mice predominately exist below 0.1 Hz. A representative power spectrum for a pixel's time trace before and after processing is shown in Fig. S9. After filtering, each pixel's time series was resampled from 30 Hz to 1 Hz for further analysis. The time traces of all pixels defined as brain were averaged to create a global brain signal. This global signal was regressed from every pixel's time trace to remove global sources of variance.

Using the atlas as a reference, seed locations were chosen at coordinates expected to correspond to the left and right visual, motor, somatosensory, frontal, cingulate, and retrosplenial cortices as well as the right and left superior colliculi and olfactory bulbs. A 0.5 mm diameter circle at each seed location was averaged to create a seed time trace. These seed traces were correlated against every other brain pixel to create functional connectivity maps. Because seed-based methods are dependent on the seed location, we also used seed-independent methods for determining connectivity patterns. The time traces in every pixel were correlated against every other pixel to create an *N*×*N* connectivity matrix (where *N* is the number of pixels defined as brain). This matrix contains all the functional connectivity information that could be gained from seed-based analysis, but has too much data to examine all at once. Taking the SVD of this matrix will yield an ordered set of orthogonal singular vectors that represent the spatial connectivity patterns. The associated singular values indicate the extent to which a particular singular vector contributes to the total variance in the data. The first few singular vectors thus demonstrate the most dominant connectivity patterns.

### Iterative Parcellation

With the goal of regenerating the atlas divisions in a data-driven manner, we parcellated the brain into functional regions using the resting-state brain signals and an iterative strategy. Similar methodology has been used to parcellate the human cingulate cortex on the basis of anatomical connectivity assessed by diffusion tensor tractography[65]. An initial assumption about the organization of the neuroarchitecture can be refined with a method consisting of two steps: (1) updated time traces are found for each parcel by averaging over all pixels in each parcel, (2) updated spatial arrangements are found for each parcel by calculating the *r*-values between the time traces for each pixel and each parcel (constructing a cross correlation matrix), and then assigning every pixel to the parcel with which it had the highest correlation coefficient. New averaged time traces are calculated for each updated parcel spatial arrangement (a return to Step 1). This cycle is repeated until no pixel changes regions from one cycle to the next. If at any point in the iterative scheme, a parcel had fewer than ten pixels (0.04 mm^2^), it was eliminated from the analysis, thus preventing the development of overly small parcels (a pixel will have the highest possible correlation coefficient with a parcel consisting solely of itself).

Results obtained by iterative techniques potentially depend on the details of initialization. Accordingly, we explored three initial parcellation conditions. The first initial condition was derived from the first ten singular modes of the connectivity matrix (after SVD) with pixels assigned to whichever singular vector with which they had the highest positive or negative coefficient. The pixels that had high positive or negative coefficients with a given parcel were then split into two different parcels, yielding twenty total parcels. While a relatively large amount of high-frequency noise is present, this initial parcellation shows the expected structure of cingulate, retrosplenial, motor, somatosensory, visual and superior colliculus (Fig. S10a). Second, we used the seeds defined above for the seed-based maps that were chosen from expected anatomy (Fig. S10b). Third, the 128×128 pixel map was divided into 25×25 pixel squares. Pixels outside of the brain were eliminated from these regions and the remaining full or partial squares were then used as initial seed regions (Fig. S10c). The first and third methods are thus completely data-driven.

Once we had stable parcellations, we investigated the network membership of the obtained regions (the numerical labels assigned to each region being completely arbitrary) using a clustering algorithm. First, we correlated every parcel against every other parcel to create a parcel-to-parcel correlation matrix. Clustering was then performed using a linkage function (Matlab™) with the distance between any two regions defined as 1-*r* (where *r* is the correlation coefficient). These clusters then were used to define functional connectivity networks and assign putative functional borders.

## Supporting Information

Figure S1 **Seed-based fcOIS correlation maps for all mice.** Seed positions and sizes are shown with black circles. The scale for all correlation maps is from *r* = −1 to 1. Maps are shown overlaid on the “white light” image of the brain.
(EPS)

Figure S2 **Correlation maps of the auditory seeds placed in Mouse 2 and Mouse 5.** Seed positions and sizes are shown with black circles. The scale for all correlation maps is from *r* = −1 to 1. Maps are shown overlaid on the “white light” image of the brain.
(EPS)

Figure S3 **Correlation map from two consecutive fifteen minute scans in the same mouse.** Note the similarity in the bilateral patterns for all seed locations in the full scan compared with the two scan halves.
(EPS)

Figure S4 **Correlation map in the same mouse imaged on two consecutive days.**
(EPS)

Figure S5 **Singular value decomposition (SVD) of the full-field correlation matrix.** The first four singular vectors strongly corroborate the seed-based correlation analysis: 1) a very strong frontal/cingulate network that anticorrelates with the bilateral sensory areas, 2) the bilateral retrosplenial cortex that anticorrelates with the sensory areas, (3) the visual areas and the superior colliculus, and (4) more medial motor areas.
(EPS)

Figure S6 **Parcellation results of Mouse 1 using three different initial conditions.** The iterative parcellation procedure converges to very similar results independent on the initial conditions. The final parcellations are compared to a histological atlas (initial conditions shown in Fig. S10 and the labeled atlas shown in Fig S8)
(EPS)

Figure S7 **Parcellations from two consecutive fifteen minute scans in the same mouse.**
(EPS)

Figure S8 **Manually constructed atlas.** Atlas of the locations of cortical functional regions (as viewed from a superior projection of the convexity) constructed using the Paxinos histological atlas [37].
(EPS)

Figure S9 **Representative resting-state power spectrum of a seed time trace in an anesthetized mouse.** The upper plot shows spectral power of raw data (ΔHbO_2_) with a peak at 2.5 Hz (pulse). Data are filtered (0.009 Hz–0.08 Hz), regressed, and resampled (1 Hz) before correlation analysis. The spectral data of the processed signal is shown in the lower plot.
(EPS)

Figure S10 **Initial conditions used for the parcellation procedure.** a) The first initial condition was derived from the first ten singular modes of the connectivity matrix (after SVD) with pixels assigned to whichever singular vector with which they had the highest positive or negative coefficient. While high-frequency is noise present, this initial parcellation shows the expected structure of cingulate, retrosplenial, motor, somatosensory, visual and superior colliculus. b) The seeds chosen from expected anatomy. c) Seeds from a square tiling of the field-of-view. The 128×128 pixel image was divided into 25×25 pixel squares with pixels outside of the brain eliminated. The remaining full or partial squares were then used as initial seed regions. The first and third methods are thus completely data-driven.
(EPS)
